# Global Burden of Upper Airway Infections: Epidemiology, Current Challenges and Future Perspectives

**DOI:** 10.3390/idr18040089

**Published:** 2026-08-18

**Authors:** Pierre Guarino, Francesco Chiari, Luigi La Via, Andrea Marino, Jerome Rene Lechien, Mario Lentini, Salvatore Lavalle, Giuseppe Nunnari, Salvatore Maira, Carmelo Giancarlo Botto, Salvatore Ferlito, Antonino Maniaci

**Affiliations:** 1 Otolaryngology Head and Neck Unit—“Santo Spirito” Hospital, 65124 Pescara, Italy; pierreguarino@hotmail.com (P.G.); francesco.chiari.med@gmail.com (F.C.); 2 Department of Innovative Technologies in Medicine & Dentistry, University “G. d’Annunzio” Chieti-Pescara, 65121 Pescara, Italy; 3 Department of Anaesthesia and Intensive Care, University Hospital Policlinico-San Marco, 24046 Catania, Italy; luigilavia7@gmail.com; 4 Unit of Infectious Diseases, Department of Clinical and Experimental Medicine, ARNAS Garibaldi Hospital, University of Catania, 95123 Catania, Italy; andrea.marino@unict.it (A.M.); giuseppe.nunnari1@unict.it (G.N.); 5 Research Study Group of the Young-Otolaryngologists of the International Federations of Oto-Rhino-Laryngological Societies (YO-IFOS), 75000 Paris, France; jerome.lechien@umons.ac.be (J.R.L.); antonino.maniaci@unikore.it (A.M.); 6 Department of Medicine and Surgery, University of Enna “Kore”, 94100 Enna, Italy; mario.lentini@unikore.it (M.L.); salvatore.lavalle@unikore.it (S.L.); salvatore.maira@unikore.it (S.M.); carmelogiancarlo.botto@asp.rg.it (C.G.B.); 7 Department of Medical, Surgical and Advanced Technology G.F. Ingrassia, University of Catania, 95123 Catania, Italy

**Keywords:** upper respiratory infections, global health, antimicrobial resistance, vaccination, public health, COVID-19

## Abstract

Background: Upper airway tract infections (UATIs) are among the most common infectious diseases worldwide, accounting for an estimated 12.8 billion episodes annually and more than 8 million disability-adjusted life years (DALYs). Despite their generally self-limiting nature, their cumulative clinical, socioeconomic, and public health burden remains substantial, particularly in children, older adults, and low- and middle-income countries (LMICs). Methods: We performed a structured narrative review of the peer-reviewed literature and reports from major international health organizations to summarize the current evidence on the epidemiology, etiology, clinical impact, socioeconomic burden, prevention strategies, and future challenges associated with UATIs. Particular attention was given to the influence of antimicrobial resistance, vaccination policies, and lessons learned from the COVID-19 pandemic. Results: UATIs remain one of the leading causes of healthcare utilization worldwide. Children experience the highest incidence, averaging 6–8 episodes annually, whereas vulnerable populations are at increased risk of complications and hospitalization. Marked geographical disparities persist, with LMICs experiencing a disproportionate burden due to limited healthcare access, lower vaccination coverage, and higher complication rates. Inappropriate antibiotic prescribing continues to accelerate antimicrobial resistance, while the COVID-19 pandemic profoundly altered the epidemiology of respiratory infections and demonstrated the effectiveness of non-pharmaceutical interventions. Advances in vaccination, antimicrobial stewardship, rapid diagnostics, and novel therapeutic strategies offer promising opportunities to reduce disease burden. Conclusions: Reducing the global burden of UATIs requires integrated public health strategies combining equitable vaccine access, effective antimicrobial stewardship, strengthened healthcare systems, and sustained surveillance. Lessons learned from the COVID-19 pandemic provide a unique opportunity to improve preparedness for future respiratory outbreaks while addressing persistent regional inequalities in prevention and care.

## 1. Introduction

Upper respiratory tract infections (URTIs), herein referred to as upper airway tract infections (UATIs), are among the most common acute infectious diseases worldwide, affecting the nasal cavity, paranasal sinuses, pharynx, and larynx [1,2]. The clinical spectrum of UATIs encompasses rhinitis (common cold), sinusitis, pharyngitis (e.g., sore throat and tonsillitis), and laryngitis. While most cases are self-limiting and mild, some infections can progress to more severe conditions, such as epiglottitis [1,3]. The etiological landscape of UATIs is predominantly viral, with approximately 90% caused by viruses, and over 200 respiratory viral types identified in the human upper airway to date [4]. Bacterial pathogens, such as Streptococcus pyogenes (Group A Streptococcus), are less frequent but may lead to significant complications, particularly in cases of pharyngitis [5]. Historically, UATIs have contributed to considerable morbidity and mortality. Diphtheria, a toxin-mediated upper airway infection, exemplifies this burden, reportedly causing over 206,000 cases and 15,000 deaths in the United States alone in 1921, prior to widespread vaccination efforts [6,7]. In the contemporary era, despite advances in medical care, UATIs remain the most common acute illnesses worldwide, accounting for a substantial number of outpatient visits and healthcare encounters [4]. According to the Global Burden of Disease (GBD) Study 2021, an estimated 12.8 billion new cases of upper respiratory infections occurred globally (excluding COVID-19), corresponding to an incidence of approximately 162,000 cases per 100,000 population per year [8]. Although mortality remains low—around 0.2 deaths per 100,000 individuals annually—the cumulative health impact is significant, with an estimated 8.16 million disability-adjusted life years (DALYs) lost globally due to UATIs and related complications such as otitis media [8]. The burden of UATIs is unevenly distributed across age groups and socioeconomic regions. Young children are particularly affected: in 2021, the highest incidence was observed in children under two years of age, with the greatest number of episodes reported in those aged five to nine years [8]. Typically, a child experiences 6–8 UATI episodes per year—approximately four times the frequency observed in adults [1]. These infections substantially affect quality of life, school attendance, and parental productivity, imposing a secondary societal and economic burden [9]. In adults, especially those with chronic illnesses or immunosuppression, UATIs can trigger complications or exacerbate underlying conditions. Notably, low- and middle-income countries (LMICs) bear a disproportionate share of both morbidity and mortality, with inadequate access to timely diagnosis and treatment increasing the risk of complications or progression to lower respiratory tract infections [10]. For instance, untreated streptococcal pharyngitis in LMICs remains a leading precursor to rheumatic fever and rheumatic heart disease [5]. These disparities reflect differences in vaccination coverage, healthcare accessibility, socioeconomic conditions, and antimicrobial stewardship programs. Although often perceived as minor illnesses at the individual level, UATIs exact a substantial cumulative toll. In the United States (US) alone, the estimated annual economic impact—including direct healthcare costs and lost productivity—is approximately $40 billion [4]. This burden is compounded by the widespread and often inappropriate use of antibiotics for presumed bacterial UATIs, despite most being viral in origin, which contributes to the global threat of antimicrobial resistance [5]. Studies report that 30–40% of uncomplicated UATIs, such as the common cold, are inappropriately treated with antibiotics [11]. Given their high frequency, preventability, and potential for complications, even modest improvements in UATI prevention and management could yield considerable public health benefits. Consequently, global health authorities underscore the importance of comprehensive surveillance, vaccine development, public education, and antimicrobial stewardship programs to reduce the impact of these infections [12].

This narrative review provides a comprehensive overview of the global burden of UATIs by integrating current evidence on epidemiology, clinical impact, socioeconomic consequences, prevention strategies, antimicrobial stewardship, and lessons learned from the COVID-19 pandemic. Furthermore, it discusses current knowledge gaps and future perspectives to support evidence-based public health strategies aimed at reducing the worldwide burden of upper airway infections.

## 2. Materials and Methods

### 2.1. Study Design

This study was designed as a structured narrative review aimed at providing a comprehensive and critical overview of the current evidence on the global burden, epidemiology, clinical impact, prevention strategies, and future challenges of upper airway tract infections (UATIs). A narrative approach was selected because of the broad and multidisciplinary nature of the topic, allowing the integration of epidemiological evidence, clinical studies, public health reports, and international policy documents that could not be adequately synthesized through a conventional systematic review. The review incorporates perspectives from otolaryngology, infectious diseases, epidemiology, microbiology, and public health to provide a balanced and clinically relevant overview of this rapidly evolving field.

### 2.2. Literature Search

A structured literature search was performed between May and July 2025 using four major bibliographic databases: PubMed, Scopus, Web of Science, and Google Scholar. The search included studies published from January 2000 to July 2025, with particular emphasis on high-quality evidence published within the last decade, while landmark older studies were included when considered historically or scientifically relevant. Search strategies combined free-text keywords and, where applicable, controlled vocabulary (MeSH terms), including “upper respiratory tract infections”, “upper airway tract infections”, “UATI”, “global burden”, “epidemiology”, “DALYs”, “antimicrobial resistance”, “antibiotic stewardship”, “vaccination”, “influenza”, “respiratory syncytial virus”, “Streptococcus pyogenes”, and “COVID-19”. Only English-language studies involving human subjects were considered. In addition, the grey literature and official reports from international organizations, including the World Health Organization (WHO), the Centers for Disease Control and Prevention (CDC), Gavi, and the United Nations Children’s Fund (UNICEF), were reviewed to obtain the most up-to-date epidemiological data and public health recommendations. The reference lists of key publications were also screened to identify additional relevant sources.

### 2.3. Study Selection, Data Extraction and Evidence Synthesis

Studies were considered eligible if they provided information on the epidemiology, incidence, prevalence, etiology, clinical manifestations, socioeconomic burden, prevention strategies, antimicrobial resistance, vaccination policies, or public health management of UATIs. Studies evaluating the impact of the COVID-19 pandemic on respiratory infection epidemiology and healthcare organization were also included. Articles focusing exclusively on lower respiratory tract infections without relevant discussion of upper airway diseases, non-human studies, non-English publications, conference abstracts, and unpublished or non-peer-reviewed reports were excluded.

All potentially relevant publications were independently screened and evaluated by at least two authors with expertise in otolaryngology, infectious diseases, epidemiology, and public health. Any discrepancies regarding study eligibility or interpretation were resolved through discussion and consensus.

**Figure 1:**
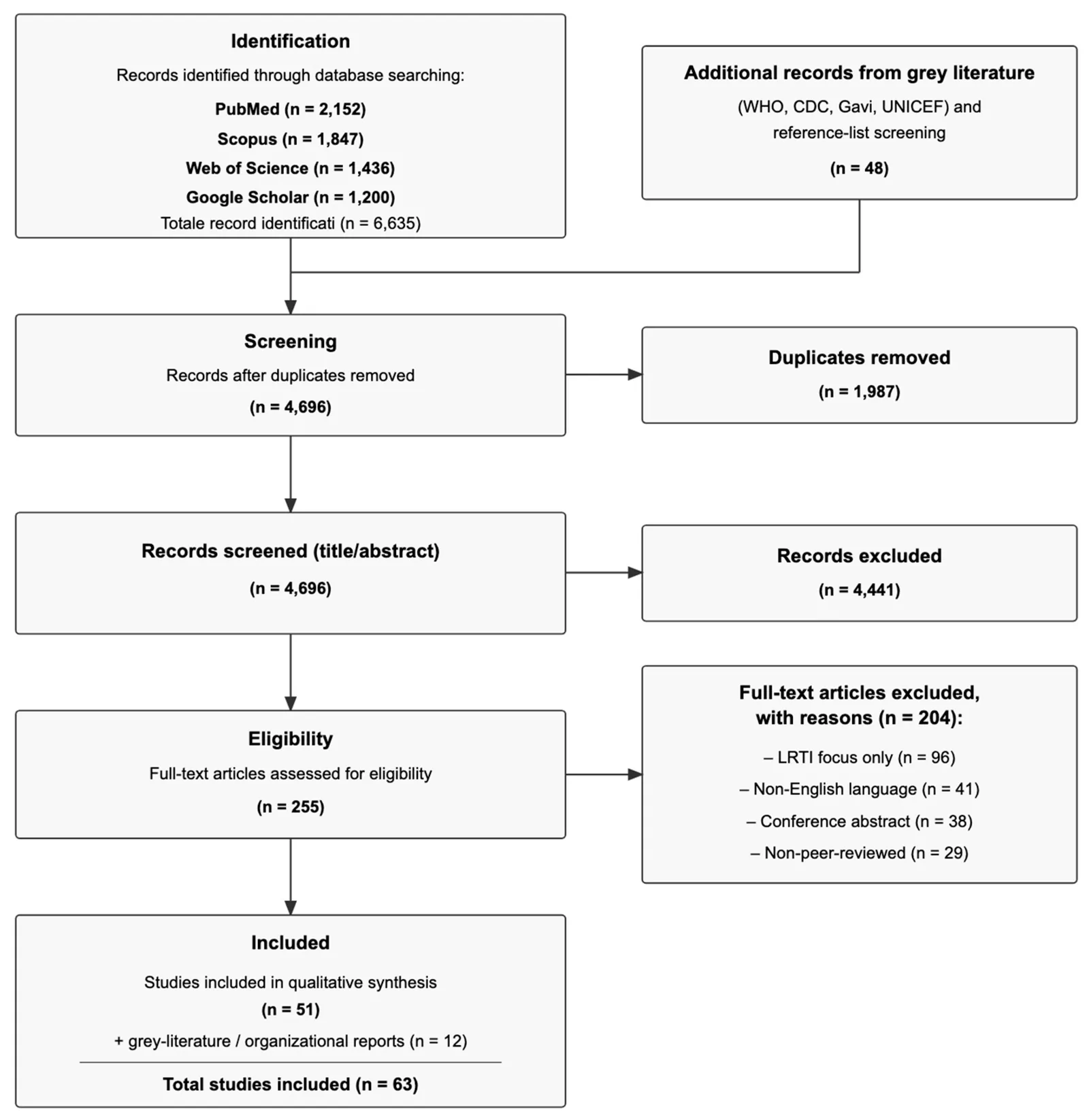
Literature search protocol.

Data were extracted qualitatively and synthesized according to predefined thematic domains, including epidemiology, etiology and pathophysiology, age-related disease burden, socioeconomic impact, health disparities, antimicrobial stewardship, vaccination strategies, pandemic preparedness, and future perspectives. Whenever appropriate, epidemiological findings were compared and integrated with data from the GBD Study and international surveillance systems to improve consistency, contextualization, and comparability across different geographical regions and healthcare settings. To improve transparency, as requested during peer review, the study-selection workflow is now summarized in a flow diagram, reporting the number of records identified through each database, duplicates removed, records screened by title/abstract, full-text articles assessed for eligibility, and studies ultimately retained for qualitative synthesis, together with the principal reasons for exclusion (non-UATI focus, non-English language, conference abstracts, or non-peer-reviewed material) (Figure 1).

### 2.4. Methodological Limitations and Critical Appraisal

As a narrative rather than a systematic review, this work does not include a formal risk-of-bias assessment or meta-analytic pooling of effect estimates; findings should therefore be interpreted as a qualitative, critically appraised synthesis rather than a quantitative summary of pooled evidence. The 2000–2025 search window was intentionally broad to capture both landmark epidemiological and microbiological studies and the substantial body of literature published after 2020, when the COVID-19 pandemic markedly altered the epidemiology of respiratory pathogens; in weighting the evidence, however, we prioritized studies published within the last decade, and studies predating 2015 were retained only when of continued historical or methodological relevance, as clarified in Section 2.2.

Several sources of uncertainty affect the estimates discussed throughout this review. First, GBD estimates rely on modelled projections combining heterogeneous data sources—including verbal-autopsy data, insurance claims, and syndromic surveillance—and are subject to substantial uncertainty, particularly in regions with limited vital registration, such as many LMICs [8,9]. Second, reported antibiotic prescribing rates vary considerably with case definitions, healthcare-seeking behavior, and diagnostic capacity; prescribing estimates derived from well-resourced ambulatory-care networks may not be generalizable to LMIC primary care, where self-medication and informal antibiotic dispensing are common but poorly captured by existing surveillance [11,12,13,14]. Third, surveillance infrastructure quality differs substantially between regions: fewer than half of WHO Member States currently report complete data to the Global Antimicrobial Resistance and Use Surveillance System (GLASS), and LMIC-specific data on UATI epidemiology, economic burden, and antimicrobial resistance remain comparatively scarce relative to high-income settings [13]. These limitations do not undermine the overall conclusions of this review but underscore the need for cautious interpretation of absolute figures and for expanded, standardized surveillance in underrepresented regions.

## 3. Epidemiology of Upper Airway Tract Infections

UATIs rank among the most prevalent acute illnesses worldwide, with notable variation in incidence across age groups, geographic regions, and seasons. Their epidemiology reflects a complex interplay of viral predominance, environmental triggers, and host-related vulnerabilities, collectively driving substantial clinical and economic burdens.

### 3.1. Global Prevalence and Incidence Rates

UATIs remain among the most prevalent infectious diseases worldwide, representing one of the leading causes of primary healthcare consultations and outpatient visits. As reported in the Introduction, this corresponds to an estimated 12.8 billion annual episodes globally [8]. Although mortality is relatively low compared with lower respiratory tract infections, the exceptionally high incidence translates into a considerable cumulative burden on healthcare systems worldwide.

The epidemiology of UATIs is characterized by substantial geographical and temporal variability, reflecting differences in climate, population density, socioeconomic conditions, vaccination coverage, healthcare accessibility, and circulating respiratory pathogens. Seasonal epidemics occur regularly in both community and institutional settings, particularly during colder months in temperate regions, while tropical areas often exhibit less pronounced but more prolonged transmission patterns [15,16].

Given their extremely high frequency, even small reductions in incidence through vaccination, infection prevention measures, and antimicrobial stewardship could produce substantial public health benefits worldwide.

### 3.2. Regional and Socioeconomic Variations

Marked regional and socioeconomic disparities characterize the global burden of UATIs, with LMICs experiencing substantially higher morbidity and disability than high-income countries. LMICs experience a disproportionately higher impact due to limited healthcare access, crowded living conditions, and lower vaccination coverage. According to a 2024 GBD analysis, approximately 8 million DALYs are lost annually due to upper respiratory infections and related complications, such as otitis media, with the highest burden observed in countries with low socio-demographic indices [8]. These DALYs encompass both years lost to premature mortality and years lived with disability, particularly in populations lacking timely access to antibiotics or supportive care [8]. In South America, acute respiratory infections alone contribute to the loss of nearly 465,000 productive life years each year, resulting in an estimated economic toll of $835 million to $2 billion [17].

A more granular analysis of the GBD 2021 estimates highlights how the composition of this burden differs between economic settings. In high-income countries, DALYs attributable to UATIs are overwhelmingly driven by years lived with disability (YLD) rather than years of life lost (YLL), reflecting low case-fatality but high prevalence and symptom burden; conversely, in low-income and lower-middle-income countries, a proportionally larger share of DALYs derives from YLL, reflecting higher case-fatality among young children with complications such as severe otitis media, mastoiditis, or progression to lower respiratory tract infection [8]. This divergence indicates that, while UATIs impose a considerable quality-of-life burden in high-income settings, in LMICs they retain a more classical infectious-disease mortality signature—implying that policy priorities should differ accordingly: health-related quality-of-life optimization and symptomatic care in high-income countries versus mortality prevention through timely diagnosis, antibiotic access, and vaccination in LMICs.

### 3.3. Age-Related Differences

Children, especially those under five years, exhibit the highest incidence of UATIs, frequently experiencing six to eight infections annually—approximately four times the rate observed in healthy adults [2]. These recurrent infections disrupt family routines and contribute substantially to pediatric outpatient visits and school absenteeism. In adults, while UATIs are generally milder, certain populations—including older adults and individuals with chronic pulmonary conditions—remain at risk for more severe manifestations and complications [2]. Health-related quality of life (HRQoL) can be significantly impaired during acute episodes in both children and adults, with adults reporting reductions in physical functioning, vitality, and social engagement comparable to those observed in patients with osteoarthritis or mild depression [2,18]. Evidence regarding sex-related differences in UATI epidemiology remains limited. Although incidence is generally comparable between males and females, biological, hormonal, and behavioral factors may influence susceptibility, healthcare utilization, and disease severity in selected populations, warranting further investigation.

### 3.4. Seasonal and Environmental Influences

The epidemiology of UATIs is strongly influenced by seasonal and environmental factors, with marked temporal variations observed across different geographical regions. These seasonal fluctuations often lead to predictable surges in healthcare utilization and absenteeism. In LMICs, however, seasonality may be influenced by local environmental factors, including monsoon patterns and indoor air quality. Additional exposures such as passive smoke inhalation, poor ventilation, and air pollution further increase susceptibility, particularly in children [15]. Behavioral factors—such as school attendance, workplace density, and hygiene practices—also modulate transmission dynamics [16].

Climate change and global demographic ageing are increasingly recognized as modifiers of UATI epidemiology. Rising ambient temperatures, altered precipitation patterns, and more frequent extreme weather events are shifting the seasonality and geographic range of several respiratory pathogens, while worsening air quality from wildfires and pollution may further increase susceptibility to upper airway infection and its complications [15,16]. In parallel, the demographic shift toward older populations in many regions is expanding the pool of individuals with immunosenescence and multimorbidity who are at elevated risk of severe or complicated UATIs, suggesting that future surveillance and prevention strategies will need to account jointly for climate-related and demographic drivers of disease burden.

## 4. Etiology and Pathophysiology

### 4.1. Common Viral Pathogens

Viruses account for approximately 90% of UATIs, with human rhinoviruses (HRVs), influenza viruses, respiratory syncytial virus (RSV), seasonal coronaviruses, adenoviruses, parainfluenza viruses, human metapneumovirus, and severe acute respiratory syndrome coronavirus 2 (SARS-CoV-2) representing the principal causative agents [19]. HRVs remain the leading cause of the common cold, comprising more than 160 serotypes and exhibiting remarkable genetic diversity, which contributes to recurrent infections throughout life [19]. Although typically associated with mild disease, HRV infections can precipitate asthma exacerbations, worsen chronic respiratory disorders, and prolong symptom duration, particularly in susceptible individuals [20].

Influenza A and B viruses remain major contributors to the global burden of seasonal respiratory infections because of their capacity for antigenic drift and occasional antigenic shift, leading to annual epidemics and periodic pandemics [21,22]. RSV is increasingly recognized as an important pathogen not only in young children but also in older adults and immunocompromised patients, where it is associated with significant morbidity and healthcare utilization.

SARS-CoV-2 has profoundly reshaped the epidemiology of respiratory infections. Beyond its direct clinical impact, the widespread implementation of non-pharmaceutical interventions—including masking, physical distancing, and improved hand hygiene—resulted in an unprecedented reduction in the circulation of influenza and RSV, whereas rhinoviruses continued to circulate, highlighting important differences in viral transmission dynamics and ecological interactions among respiratory pathogens [23].

### 4.2. Bacterial Pathogens and Antibiotic Resistance Trends

Although viral infections predominate, bacterial pathogens contribute notably to UATIs, particularly in cases of pharyngitis and sinusitis. Group A Streptococcus pyogenes accounts for up to 15% of acute pharyngitis in children, while Streptococcus pneumoniae, Haemophilus influenzae, and Corynebacterium diphtheriae are responsible for a smaller proportion of bacterial UATIs [5]. Despite the viral etiology, antibiotics are frequently overprescribed, with up to 40% of viral UATIs treated unnecessarily, promoting antimicrobial resistance (AMR). Global antibiotic consumption rose approximately 16% between 2016 and 2023, paralleled by increasing AMR, particularly in LMICs with surveillance gaps [13]. Nasopharyngeal and oropharyngeal microbiomes serve as reservoirs for resistance genes; adults exhibit higher expression of AMR genes than children, potentially facilitating transmission and complicating management of both upper and lower respiratory infections [5]. Infections with less common pathogens, such as oropharyngeal syphilis, further complicate management, as misdiagnosis or inappropriate antibiotic use can drive AMR [24]. Although invasive GAS disease is rare in neonates, a recent systematic review reported a mortality rate of approximately 14% and heterogeneous clinical presentation among neonatal invasive GAS cases, distinguishing early- and late-onset disease patterns and underscoring the importance of prompt recognition and treatment in this vulnerable population [25].

### 4.3. Host Factors and Comorbidities Influencing Disease Burden

Host-related factors play a pivotal role in determining susceptibility, clinical severity, and outcomes of UATIs. Young children are particularly vulnerable because of immune system immaturity, smaller airway anatomy, and frequent exposure to respiratory pathogens in daycare and school settings, resulting in higher infection rates and an increased risk of complications [2].

In adults, several chronic conditions—including asthma, chronic obstructive pulmonary disease, diabetes mellitus, cardiovascular disease, obesity, and immunosuppression—predispose patients to more severe infections and prolonged recovery. Human rhinovirus infections are a well-recognized trigger of acute asthma exacerbations and may worsen underlying chronic respiratory diseases [1].

Older adults are particularly susceptible because of immunosenescence, multimorbidity, and age-related alterations of the respiratory microbiome, which may contribute to increased colonization by resistant microorganisms and a higher burden of antimicrobial resistance genes [5].

Beyond host characteristics, interactions among respiratory viruses may also influence disease dynamics. Experimental and epidemiological studies suggest that prior infection with human rhinovirus or influenza viruses may transiently inhibit subsequent SARS-CoV-2 replication through interferon-mediated antiviral responses, whereas the sequence of viral exposures may influence both transmissibility and clinical severity [1]. Beyond direct infection, respiratory pathogens implicated in UATIs—including Mycoplasma pneumoniae, influenza viruses, SARS-CoV-2, adenovirus, and RSV—have also been linked to post-infectious immune-mediated complications in children, such as reactive infectious mucocutaneous eruption, in which exaggerated innate and adaptive immune responses, rather than pathogen persistence, drive severe mucosal injury, illustrating the broader immunological consequences upper airway pathogens can trigger beyond the acute respiratory illness itself [14].

Together, host susceptibility, underlying comorbidities, microbial ecology, and viral interactions contribute to the heterogeneous clinical presentation and global burden of UATIs, emphasizing the need for individualized prevention strategies and targeted public health interventions.

## 5. Clinical and Socioeconomic Impact

### 5.1. Disease Burden in Children, Adults, and the Elderly

The clinical and socioeconomic burden of UATIs varies considerably across different age groups. Children, particularly those younger than five years, experience the highest incidence and DALYs, reflecting both frequent infections and an increased risk of complications such as acute otitis media and rhinosinusitis [2,8]. Recurrent respiratory infections substantially impair physical, emotional, social, and school functioning, resulting in measurable reductions in health-related quality of life for both children and their families [9].

In adults, UATIs are generally self-limiting but remain one of the leading causes of primary care consultations, temporary work absenteeism, and productivity losses, generating a considerable healthcare and socioeconomic burden.

Older adults represent a particularly vulnerable population because of immunosenescence, frailty, and multiple comorbidities. In this age group, respiratory infections are associated with higher hospitalization rates, increased healthcare costs, and greater morbidity than in younger adults. RSV, traditionally considered a pediatric pathogen, is now recognized as an important cause of severe respiratory disease in older adults and immunocompromised individuals, with hospitalization rates and clinical outcomes comparable to those associated with seasonal influenza [26].

When UATIs progress to lower respiratory tract involvement, the resulting community-acquired pneumonia can require hospitalization and carries a clinical profile that varies by causative pathogen. A recent case–control study of adult inpatients with Legionella pneumophila pneumonia found that, compared with pneumonia of other etiologies, Legionella cases were associated with a higher comorbidity burden and were more likely to present with bilateral pulmonary infiltrates and mediastinal or hilar lymphadenopathy on imaging—findings that may assist clinicians in the early recognition of this often-underdiagnosed cause of severe respiratory infection [27]. Together with pneumococcal-, influenza-, and RSV-associated pneumonia, Legionella infection illustrates the continuum between upper airway disease and life-threatening lower respiratory tract infection, reinforcing the clinical relevance of prompt recognition, targeted diagnostics, and appropriate escalation of care.

### 5.2. Economic Costs: Direct and Indirect Burden

UATIs generate a substantial economic burden through direct healthcare expenditures and indirect societal costs, including absenteeism, presenteeism, reduced productivity, and increased utilization of healthcare resources. Given their exceptionally high incidence, even self-limiting infections result in considerable cumulative economic consequences.

In the United States, non-influenza viral respiratory infections have been estimated to generate approximately US$40 billion annually in direct medical costs and productivity losses, accounting for more than 150 million lost workdays each year. In addition, expenditure on over-the-counter medications exceeds US$2.9 billion annually [4]. The common cold alone is responsible for approximately 75–100 million physician visits per year, generating an estimated US$7.7 billion in healthcare costs and more than US$20 billion in productivity losses [4].

Similar economic challenges have been reported in Europe and other high-income regions, where respiratory infections—particularly among older adults—are associated with increased hospitalizations, outpatient visits, and healthcare expenditures. These findings underscore that the burden of UATIs extends well beyond their generally benign clinical course, representing an important challenge for healthcare systems, employers, and society as a whole [17].

Data from South Asia, sub-Saharan Africa, and Southeast Asia—regions where UATIs impose their greatest absolute burden—remain comparatively limited, but available evidence indicates that the economic impact on households can be disproportionately severe. A multicountry analysis of influenza-like illness and acute respiratory infection costs across 11 LMICs in Africa, Asia, and Latin America found that out-of-pocket expenditure represented the highest proportion of annual household income among the lowest income quintile in the large majority of settings studied, with median out-of-pocket costs for hospitalized illness exceeding 10% of annual household income—the conventional threshold for catastrophic health expenditure—among the general population and children in Kenya and among older adults and patients with chronic disease in China [28]. Comparable catastrophic-expenditure patterns have been documented across South and Southeast Asia, where limited health-insurance coverage and heavy reliance on private out-of-pocket care compound the economic consequences of respiratory illness. Unlike in high-income countries, where the economic burden of UATIs is dominated by productivity losses, in LMICs a substantial share of the burden falls directly on already vulnerable households, reinforcing the need for expanded financial risk protection—including health insurance and subsidized primary care—alongside preventive interventions such as vaccination and improved sanitation.

### 5.3. Impact on Quality of Life and Work Productivity

Beyond their clinical manifestations, UATIs substantially affect HRQoL, educational performance, workplace productivity, and overall societal functioning. Although most infections are self-limiting, their exceptionally high incidence translates into a considerable cumulative impact on patients, caregivers, and healthcare systems.

In children, recurrent respiratory infections interfere with physical well-being, social interactions, school attendance, and educational performance, while simultaneously increasing caregiver burden and parental work absenteeism [2]. These effects extend beyond the acute illness, contributing to measurable reductions in family quality of life.

Among working-age adults, UATIs are a major cause of both absenteeism and presenteeism, with many individuals continuing to work despite symptoms, resulting in reduced productivity and impaired work performance. In many settings, productivity losses attributable to presenteeism exceed those associated with sick leave alone [4].

In older adults, recurrent respiratory infections—particularly those caused by RSV—are associated with worsening functional status, reduced HRQoL, increased healthcare utilization, and greater dependence on caregivers, placing an additional burden on families and healthcare systems [4].

### 5.4. Burden in LMICs

The burden of UATIs is disproportionately high in LMICs due to limited healthcare access, inadequate sanitation, and socioeconomic challenges. Overcrowded living conditions and poor hygiene exacerbate transmission and complications; insufficient sanitation alone contributes to hundreds of thousands of respiratory deaths annually [8]. Disability due to upper respiratory infections and related conditions such as otitis media peaks in low socio-demographic index regions, resulting in millions of DALYs lost [8]. HRQoL assessments tailored to children in these settings remain scarce, complicating both burden evaluation and the design of targeted interventions [2]. High incidence of bacterial complications, coupled with limited access to timely medical care, magnifies both health and economic consequences, underscoring respiratory infections as a critical public health priority in resource-limited contexts.

### 5.5. Clinical Warning Signs Warranting Further Evaluation

Most UATIs can be managed conservatively in the outpatient setting; however, clinicians should remain alert to warning signs that warrant further investigation or hospital referral. These include high or persistent fever beyond 3–5 days, respiratory distress or increased work of breathing, stridor or drooling suggestive of epiglottitis, inability to maintain oral intake or signs of dehydration, altered mental status, poor peripheral perfusion, unilateral peritonsillar swelling suggestive of abscess, neck stiffness or torticollis raising concern for retropharyngeal infection, and clinical deterioration despite appropriate symptomatic treatment—particularly in infants, older adults, and immunocompromised patients. Recognition of these features allows timely escalation of care and reduces the risk of complications associated with delayed diagnosis of evolving severe infection.

## 6. Global Health Challenges

### 6.1. Vaccination Gaps and Preventive Measures

Vaccination remains one of the most effective public health interventions for reducing the burden of UATIs, preventing complications, and limiting healthcare costs. Nevertheless, important gaps in vaccine coverage persist worldwide. Global immunization rates had already begun to plateau before the COVID-19 pandemic and subsequently declined because of disruptions to healthcare services and routine vaccination programs [6]. In 2020, an estimated 23 million children missed basic childhood immunizations—the highest number recorded in more than a decade—with the greatest impact observed in LMICs [6,7]. Although vaccination coverage has partially recovered, it remains below pre-pandemic levels in many regions, contributing to the resurgence of vaccine-preventable respiratory diseases.

Marked disparities in vaccine access continue to characterize global respiratory infection prevention. Only 34% of LMICs have national influenza vaccination policies compared with approximately 85% of high-income countries, while almost 97% of global influenza vaccine doses are administered in wealthier nations [21]. Similar inequalities exist for other respiratory vaccines, leaving vulnerable populations disproportionately exposed to preventable infections.

International initiatives have sought to address these disparities. The WHO Immunization Agenda 2030 prioritizes reducing the number of zero-dose children, whereas Gavi-supported vaccination programs have immunized more than 1.1 billion children since 2000 and are estimated to have prevented approximately 18.8 million future deaths [21]. Despite these achievements, financial constraints, logistical barriers, vaccine hesitancy, and fragile healthcare systems continue to limit equitable vaccine uptake.

These disparities differ qualitatively between economic settings. In LMICs, the principal barriers to vaccination remain structural—insufficient supply, cold-chain limitations, workforce shortages, and out-of-pocket costs—whereas in high-income countries, where supply is generally adequate, vaccine hesitancy driven by misinformation, distrust in health authorities, and pandemic-related fatigue has emerged as an increasingly important determinant of suboptimal coverage, particularly for influenza and, more recently, RSV immunization [21]. Addressing these divergent challenges requires correspondingly different strategies: continued investment in supply chains, financing, and workforce capacity in LMICs, alongside targeted risk-communication and confidence-building interventions in high-income settings.

The COVID-19 pandemic highlighted both the vulnerability and the resilience of global immunization programs. Although routine vaccination services were severely disrupted, the pandemic accelerated vaccine innovation—including mRNA technologies—and demonstrated the feasibility of rapid international collaboration through initiatives such as COVAX. Sustaining these advances and ensuring equitable vaccine access will be essential for reducing the future burden of respiratory infections worldwide [21].

### 6.2. Antimicrobial Stewardship and Antimicrobial Resistance

The misuse of antimicrobials in treating UATIs has precipitated a growing crisis of AMR. Worldwide, drug-resistant infections were responsible for an estimated 1.27 million deaths in 2019, surpassing annual deaths from human immunodeficiency virus (HIV) or malaria [12]. UATIs are mostly viral, yet antibiotics are often over-prescribed for conditions like viral bronchitis, pharyngitis, or the common cold. Studies reveal that a large fraction of antibiotic prescriptions for respiratory infections are inappropriate—for instance, in one multicenter study over 80% of adults with confirmed viral respiratory infections still received antibiotics [11]. Such overuse accelerates the emergence of resistant bacteria. In LMICs, the threat is amplified by easy over-the-counter access to antibiotics and weaker stewardship. Patients frequently purchase antibiotics without prescriptions, and clinicians, lacking diagnostic tools to distinguish viral from bacterial infection, may err on the side of antibiotic use. As a result, formerly benign UATIs can seed resistant strains of Streptococcus pneumoniae, Streptococcus pyogenes and others, complicating the treatment of more severe infections downstream.

Global health authorities have sounded the alarm on AMR as a universal threat. The WHO’s Global Action Plan on AMR (2015) and subsequent resolutions urge countries to adopt rational antibiotic use policies and strengthen surveillance [12]. One benchmark is the WHO AWaRe classification, which calls for at least 60% of national antibiotic consumption to come from the narrow-spectrum “Access” group. Most countries are far from this goal: in 2022, only about 52% of antibiotics used globally were Access antibiotics (first-line, safer drugs), with the remainder being broad-spectrum “Watch” or last-resort “Reserve” drugs. This over-reliance on broad-spectrum agents is especially notable in LMIC settings and is linked to higher resistance rates [13]. Meanwhile, surveillance of AMR in UATIs and other infections remains inadequate—less than half of countries worldwide contribute data to WHO’s GLASS monitoring system, leaving critical blind spots [13].

Pathogen-specific resistance trends further illustrate the clinical stakes of antimicrobial stewardship in UATIs. Streptococcus pyogenes has remained almost universally susceptible to penicillin worldwide for decades, and penicillin (or amoxicillin) remains the recommended first-line therapy for confirmed streptococcal pharyngitis across international guidelines [29]. However, resistance to macrolides—used as first-line therapy in penicillin-allergic patients—has been rising in several regions; recent institutional surveillance reported erythromycin resistance approaching 30% and clindamycin resistance around 28% among invasive GAS isolates, with parallel trends across parts of Europe [30]. Streptococcus pneumoniae resistance patterns also vary by region, with penicillin and macrolide non-susceptibility more prevalent in parts of Asia and southern Europe than in northern Europe or North America, reflecting differences in antibiotic consumption and pneumococcal conjugate vaccine coverage. Haemophilus influenzae beta-lactamase production, which confers amoxicillin resistance, is similarly reported in a substantial minority of isolates in several surveillance networks, necessitating beta-lactamase-stable regimens as amoxicillin-clavulanate, when H. influenzae is suspected or confirmed. These regional differences reinforce that empirical antibiotic choices for UATIs should be guided by local resistance data rather than generalized international recommendations. Outpatient antimicrobial stewardship interventions—including delayed-prescription strategies, clinician audit-and-feedback, patient-directed education, and point-of-care testing—consistently reduce unnecessary antibiotic prescribing for UATIs in primary care without compromising outcomes, and their scale-up, particularly in LMIC primary care where stewardship programs remain underdeveloped, represents a key opportunity to curb AMR at its principal driver [11,13]. Advances in rapid microbiological diagnosis represent a further promising avenue: point-of-care rapid antigen tests for group A Streptococcus, multiplex syndromic PCR panels capable of simultaneously identifying influenza viruses, RSV, SARS-CoV-2, and common bacterial pathogens, and rapid procalcitonin-guided algorithms can shorten time to etiological diagnosis, reduce unnecessary empirical antibiotic prescribing, and support timely, pathogen-targeted therapy in patients with severe or hospitalized respiratory infections. Wider implementation of these diagnostics—particularly in primary care and resource-limited settings where they remain least accessible—should be considered a stewardship priority alongside education- and policy-based interventions.

The COVID-19 pandemic has further exacerbated AMR challenges. During the early pandemic, antibiotics were widely administered to COVID-19 patients (often empirically, to cover possible bacterial co-infection), and infection control lapses in overwhelmed hospitals led to surges in drug-resistant infections [13]. For example, US hospitals reported a ≥15% increase in certain hospital-onset resistant infections in 2020 compared to pre-pandemic rates, and similar trends were observed in many regions [11]. This regression underscores how fragile gains against AMR can be. On a positive note, COVID-19 also raised public awareness about appropriate antibiotic use (with messaging that antibiotics do not treat viruses) and spurred investments in laboratory capacity that could be leveraged for AMR surveillance. Moving forward, tackling AMR in UATIs will require sustained global efforts: improving antibiotic stewardship training, expanding rapid diagnostic testing (to distinguish viral vs. bacterial infections), and continued implementation of WHO’s action plan through a One Health approach [12].

### 6.3. Health System Limitations in Addressing UATIs

Underlying health system limitations form a major barrier to managing UATIs, particularly in resource-limited settings. Many LMICs struggle with inadequate primary healthcare infrastructure—shortages of trained healthcare workers, insufficient clinics in rural areas, and user fees can all delay care-seeking for respiratory infections. Surveys indicate that in parts of sub-Saharan Africa, over 40% of caregivers delay or do not seek care for children with pneumonia symptoms due to access and cost barriers [16,31]. Such delays mean that mild upper airway infections can progress to severe illness (or lower respiratory tract involvement) before treatment is obtained. When patients do present, clinics may lack essential tools and must often treat empirically. Medicine stock-outs are another issue—inconsistent supplies of antibiotics, or of vaccines and preventive tools, hinder effective UATI management in low-resource health systems [6]. Preventive public health measures (like handwashing infrastructure, crowding reduction, indoor air quality improvement) also remain suboptimal in many HICs, allowing respiratory infections to spread easily [32].

The COVID-19 pandemic starkly exposed these health system gaps—and in some cases, created new ones. At the height of the pandemic, 90% of countries reported disruption to essential health services [33]. Routine management of UATIs suffered as outpatient clinics cut back services and patients avoided facilities. Immunization campaigns were postponed, and the management of non-COVID respiratory illnesses often fell through the cracks. Even in 2021–2022, nearly half of countries were still reporting ongoing healthcare disruptions as they struggled to restore services. Furthermore, pandemic-related economic strains have tightened health budgets in many LMICs, limiting the resources available for bolstering primary care and infection control. Health workforce limitations were also aggravated as staff were diverted to COVID-19 duties or fell ill; this reduced capacity for addressing routine infections.

Nevertheless, the pandemic response has yielded some improvements that could benefit UATI control. Notably, there was unprecedented investment in oxygen and critical care infrastructure in LMIC hospitals. The ACT-Accelerator Therapeutics pillar (led by WHO and UNICEF) mobilized hundreds of millions of dollars for the Oxygen Emergency Taskforce, delivering oxygen concentrators, cylinders, and even pressure swing adsorption plants to over 120 countries [21]. This rapid scale-up of medical oxygen availability—a key therapy for severe lower respiratory tract infections—has created a more robust platform to manage complications of UATIs like severe croup or pneumonia. These investments will continue to save lives well beyond COVID-19, strengthening health system capacity for respiratory care. Another positive development has been the expansion of telemedicine and digital health solutions. During lockdowns, telehealth platforms were used in some settings to triage and manage milder UATIs remotely, reducing unnecessary antibiotic prescriptions and maintaining care access. While digital divides mean telemedicine is not yet universally accessible, its accelerated adoption offers a blueprint for extending UATI management to underserved areas via mobile health units, apps, and call centers.

## 7. Vaccination Programs and Herd Immunity

Vaccination represents one of the most effective public health interventions for reducing the incidence, transmission, and complications of UATIs. Beyond providing direct protection to vaccinated individuals, widespread immunization generates herd immunity, interrupting pathogen circulation and protecting vulnerable populations who cannot be vaccinated or develop suboptimal immune responses. The effectiveness of this strategy has been demonstrated by historical vaccination programs. For example, Japan’s nationwide influenza vaccination campaign among schoolchildren was associated with an approximately 36% reduction in influenza-related mortality among older adults, preventing an estimated 1000 deaths annually and highlighting the profound population-level benefits of herd immunity [21]. Similarly, routine diphtheria immunization through the WHO Expanded Programme on Immunization reduced global disease incidence by more than 90% between 1980 and 2000, while widespread vaccination against Streptococcus pneumoniae and Haemophilus influenzae type b has substantially reduced invasive pneumococcal disease and upper airway complications such as acute otitis media [6,7].

The effectiveness of vaccination programs depends not only on vaccine efficacy but also on achieving sufficiently high coverage to maintain herd immunity. Although the required threshold varies according to pathogen transmissibility, maintaining high vaccination rates remains essential to prevent disease resurgence and protect high-risk populations. Persistent inequalities in vaccine access, however, continue to compromise these benefits, particularly in low- and middle-income countries (LMICs), where incomplete immunization coverage increases susceptibility to vaccine-preventable respiratory infections and their complications [21].

Recent advances in vaccine technology are expanding preventive opportunities against respiratory pathogens. The rapid development of mRNA platforms during the COVID-19 pandemic has accelerated the development of next-generation influenza and RSV vaccines, alongside recombinant protein and viral vector vaccines [6]. The approval of RSV vaccines for older adults and the introduction of long-acting monoclonal antibodies, such as nirsevimab, for infant prophylaxis represent major milestones in respiratory infection prevention. At the same time, considerable research efforts are focused on pathogens for which licensed vaccines remain unavailable, particularly Streptococcus pyogenes. Several multivalent M-protein and epitope-based vaccine candidates are currently under clinical evaluation and may substantially reduce the burden of streptococcal pharyngitis and its sequelae, especially in resource-limited settings where rheumatic fever remains endemic [6].

Maternal immunization has emerged as a particularly important strategy for RSV prevention in early infancy. In the phase 3 MATISSE trial, a single dose of the bivalent RSV prefusion F protein vaccine (RSVpreF) administered during the third trimester of pregnancy conferred 82.4% efficacy against severe RSV-associated lower respiratory illness in infants within 90 days of birth, with efficacy persisting at 70.0% through 180 days postnatally [34]. Direct comparative data indicate that passive infant immunization with the long-acting monoclonal antibody nirsevimab may confer greater protection than maternal RSVpreF vaccination: a population-based French cohort study reported a lower risk of RSV-associated hospitalization (adjusted HR 0.74) and of severe outcomes, including paediatric intensive care unit admission, among infants who received nirsevimab compared with those born to RSVpreF-vaccinated mothers [35]. These findings suggest that maternal vaccination and infant monoclonal antibody prophylaxis should be regarded as complementary rather than interchangeable strategies, with national immunization programs increasingly incorporating both, tailored to seasonal timing, gestational age at delivery, and resource availability. Beyond RSV, seasonal influenza vaccination coverage continues to fall short of WHO targets in most countries, reflecting supply constraints in LMICs and vaccine hesitancy in high-income settings (Section 6.1). Pneumococcal conjugate vaccines (PCVs) have substantially reduced invasive pneumococcal disease and, by lowering nasopharyngeal carriage, appear to reduce the burden of pneumococcal-associated acute otitis media and sinusitis, although serotype replacement remains an ongoing concern for long-term effectiveness. For Streptococcus pyogenes, no licensed vaccine currently exists despite the substantial global burden of streptococcal pharyngitis and its non-suppurative sequelae (rheumatic fever and rheumatic heart disease); several multivalent M-protein and conserved-antigen vaccine candidates are in early- to mid-stage clinical development, and their successful licensure would represent a major advance for LMICs where rheumatic heart disease remains endemic. Across all these programs, implementation barriers—vaccine affordability, cold-chain requirements, health workforce capacity, and public confidence—remain the principal determinants of real-world impact, reinforcing that technological innovation must be matched by equitable, well-resourced delivery systems.

Looking ahead, innovative preventive strategies—including intranasal vaccines capable of inducing mucosal immunity, universal influenza vaccines, and multivalent respiratory virus formulations—have the potential to further reduce the global burden of UATIs. Maximizing these benefits will require not only continued technological innovation but also equitable vaccine access, sustained public confidence, and strong international collaboration to ensure that advances in vaccine development translate into meaningful reductions in respiratory disease worldwide [6,7].

### 7.1. Public Health Interventions (Hygiene, Education, Policies)

Non-pharmaceutical public health measures remain essential for reducing the burden of UATIs, particularly where vaccine coverage or healthcare access is limited. Hygiene interventions, especially hand hygiene, significantly reduce respiratory infections. Meta-analyses have shown that handwashing promotion lowers acute respiratory infections by approximately 20–25% [36]. In LMICs, a large systematic review including 26 studies and over 160,000 participants reported a 17% reduction in overall ARI burden and a 26% decrease in upper respiratory infections following structured handwashing programs [32]. These findings highlight an important opportunity to reduce endemic respiratory disease through simple preventive measures [32].

Regular handwashing, respiratory etiquette, and public education should be continuously promoted. This is particularly important in LMICs, where 27% of people lack access to a household water supply and one-third lack soap for handwashing. Investments in water and sanitation infrastructure, combined with hygiene education, can substantially improve respiratory health [16]. School-based programs have also been shown to reduce illness-related absenteeism while encouraging lifelong healthy behaviors [16,36].

Education and mass communication campaigns remain fundamental for improving awareness of vaccination, respiratory etiquette, symptom recognition, and appropriate healthcare-seeking behavior. In both high- and low-income settings, mass media, school-based initiatives, and community outreach programs contribute to better adherence to preventive measures and reduced respiratory infection transmission [36].

The COVID-19 pandemic demonstrated that coordinated public health interventions—including mask use, physical distancing, hand hygiene, and improved ventilation—can profoundly reduce respiratory virus circulation. During 2020, influenza activity declined by 90–98% in many regions following widespread implementation of these measures [37,38]. Although such interventions are not sustainable as long-term strategies, they demonstrated the effectiveness of targeted non-pharmaceutical measures during periods of increased viral circulation.

Future public health policies should integrate improved indoor ventilation, smoke-free legislation, paid sick leave, and surveillance-guided interventions during seasonal outbreaks to reduce transmission while minimizing social disruption. Together with vaccination and antimicrobial stewardship, these measures represent key components of a comprehensive strategy to reduce the global burden of UATIs [13,39,40].

### 7.2. Novel Therapies and Research Directions

Continued innovation in therapeutics is essential to further reduce the global burden of UATIs. Although current management remains largely supportive, several antiviral agents are expanding treatment options. For influenza, next-generation drugs such as the endonuclease inhibitor baloxavir complement existing neuraminidase inhibitors, while broad-spectrum antivirals including nitazoxanide are under investigation for activity against multiple respiratory viruses [41,42].

Despite decades of research, effective therapies for HRV remain limited because of its extensive serotype diversity. Novel compounds targeting conserved viral enzymes, viral attachment, or host antiviral pathways are under development. Agents such as rupintrivir and pleconaril have shown encouraging results in reducing viral replication or shortening symptom duration, although concerns regarding efficacy and resistance have limited their clinical application [43]. Emerging strategies, including RNA interference and host-directed antivirals that enhance innate interferon responses, may provide broader protection against respiratory viruses [44]. The COVID-19 pandemic has also accelerated the development of broad-spectrum antivirals that could be rapidly deployed against emerging respiratory pathogens [45].

Modulation of the respiratory microbiome represents another promising strategy. Streptococcus salivarius K12 has been associated with reductions in recurrent streptococcal pharyngitis, viral pharyngitis, and otitis media, although further high-quality studies are needed to confirm its effectiveness [46]. Other approaches, including intranasal bacterial interference and bacteriophage-derived therapies targeting respiratory pathogens, are also under investigation [47].

Immunomodulatory strategies have recently achieved important advances. The approval of nirsevimab has significantly improved RSV prevention in infants, demonstrating approximately 75–80% efficacy in preventing RSV-related illness and hospitalization [48]. Similarly, bacterial lysates such as OM-85 have reduced recurrent respiratory infections, antibiotic use, and illness duration, particularly in children with recurrent infections [49].

Vaccine research continues to complement therapeutic innovation. Universal influenza vaccines, pan-coronavirus vaccines, vaccines targeting Streptococcus pyogenes, and novel mRNA- and mucosal-based platforms may substantially improve future UATI prevention [50,51,52].

Artificial intelligence (AI) and predictive clinical models are increasingly being explored to support the diagnosis, risk stratification, and management of respiratory infections. Machine-learning algorithms applied to clinical, laboratory, and imaging data have shown promise in differentiating viral from bacterial UATIs, predicting the risk of complications or hospitalization, and supporting antimicrobial stewardship by flagging cases in which antibiotic prescribing is unlikely to be indicated. Integration of such tools into electronic health records and point-of-care platforms could help standardize decision-making, reduce unnecessary antibiotic use, and enable earlier identification of patients at risk of severe disease, although prospective validation across diverse healthcare settings, particularly in LMICs, remains necessary before widespread clinical adoption.

## 8. Lessons Learned from the COVID-19 Pandemic

### 8.1. Impact on the Epidemiology of UATIs

The COVID-19 pandemic profoundly reshaped the epidemiology of UATIs. The widespread implementation of masking, physical distancing, school closures, and enhanced hygiene measures led to a dramatic decline in the circulation of influenza, RSV, and human metapneumovirus. During 2020–2021, non-SARS-CoV-2 respiratory infections decreased by more than 90% in many regions following the implementation of non-pharmaceutical interventions (NPIs) [38]. In contrast, rhinoviruses rapidly re-emerged, likely because of their greater environmental stability and the complex interactions among circulating respiratory viruses [38].

These unprecedented changes altered the seasonal epidemiology of respiratory viruses, highlighting both the effectiveness of NPIs in reducing transmission and the importance of maintaining robust surveillance systems to monitor viral circulation as restrictions were progressively lifted [38]. Beyond these epidemiological effects, the pandemic profoundly influenced otolaryngology practice and healthcare organization, prompting substantial changes in clinical pathways, outpatient services, and surgical activity, as documented by nationwide Italian studies [53,54,55].

### 8.2. Global Preparedness and Response

The COVID-19 pandemic exposed both the strengths and weaknesses of global preparedness for respiratory infectious diseases. The rapid implementation of non-pharmaceutical interventions, together with the unprecedented development and deployment of vaccines through initiatives such as COVAX and mRNA platforms, demonstrated the value of international collaboration and scientific innovation [56]. However, major challenges—including supply chain disruptions, misinformation, and marked vaccine inequities, with high-income countries receiving booster doses while many low-income regions lacked access to primary vaccination—highlighted persistent gaps in global health governance and equitable resource allocation [56].

In response, international efforts have focused on strengthening pandemic preparedness through initiatives such as the WHO Pandemic Agreement and other multilateral frameworks promoting data sharing, equitable access to medical countermeasures, and One Health surveillance [57]. Nevertheless, translating these commitments into effective implementation remains a major challenge. Strengthening resilient health systems, improving international coordination, and ensuring equitable access to diagnostics, vaccines, and therapeutics will be essential to improve preparedness for future respiratory outbreaks [57].

### 8.3. Implications for Future Outbreaks

The COVID-19 pandemic provided important lessons for managing future UATI outbreaks and respiratory pandemics. The rapid implementation of NPIs demonstrated their effectiveness in reducing the transmission of respiratory pathogens. However, future strategies should balance infection control with their social, economic, and educational consequences, favouring targeted and seasonal approaches over prolonged restrictions. The pandemic also accelerated the adoption of digital health technologies, including telemedicine, real-time surveillance, and digital platforms for patient triage, which can improve routine respiratory infection management and facilitate early outbreak detection.

Another major legacy is the development of rapid vaccine platforms. The “100-day challenge” has established a new benchmark, aiming to enable pathogen identification, vaccine development, manufacturing, and initial deployment within months rather than years following the emergence of a novel respiratory pathogen [58]. Achieving this objective will require sustained investment in healthcare infrastructure, laboratory capacity, workforce training, and international collaboration. Finally, the pandemic reinforced the importance of integrated One Health surveillance systems capable of monitoring human, animal, and environmental reservoirs to enable early detection and rapid containment of emerging respiratory threats [58] (Table 1).

## 9. Conclusions

UATIs remain among the most common infectious diseases worldwide, imposing a substantial clinical, socioeconomic, and public health burden, particularly among children, older adults, and populations in LMICs. Although generally self-limiting, their exceptionally high incidence continues to place considerable pressure on healthcare systems and contributes to inappropriate antibiotic use, antimicrobial resistance, and significant productivity losses.

The COVID-19 pandemic demonstrated both the vulnerability of healthcare systems and the effectiveness of coordinated public health interventions in reducing respiratory virus transmission. At the same time, advances in vaccine development, surveillance systems, digital health, and antimicrobial stewardship have created new opportunities to improve respiratory infection prevention and management.

Despite the breadth of evidence reviewed, important gaps remain. Data from LMICs are comparatively sparse for economic burden, antimicrobial resistance surveillance, and health-related quality of life, limiting the precision of global burden estimates and the ability to tailor interventions to the settings that bear the greatest disease burden. Uncertainty also persists regarding the true prevalence of inappropriate antibiotic prescribing outside well-resourced ambulatory-care networks, the comparative real-world effectiveness of maternal RSV vaccination versus infant nirsevimab prophylaxis across diverse populations, and the long-term impact of pneumococcal conjugate vaccination on UATI-specific outcomes. Future research should prioritize standardized, comparable surveillance of UATI epidemiology and antimicrobial resistance across regions, prospective evaluation of rapid diagnostic and AI-assisted decision-support tools in real-world primary care, and pragmatic trials of stewardship interventions tailored to LMIC health systems.

Reducing the global burden of UATIs will require integrated, multidisciplinary public health strategies that combine equitable vaccine access—including maternal RSV immunization, nirsevimab prophylaxis, pneumococcal and influenza vaccination, and continued investment in streptococcal vaccine development—with rational antibiotic use supported by rapid diagnostics and outpatient stewardship, strengthened primary healthcare systems, and sustained public health interventions. For clinicians, this translates into judicious use of point-of-care diagnostics, adherence to local resistance-informed prescribing guidance, and vigilance for the clinical warning signs described in Section 5.5. For policymakers and global health authorities, priorities should include expanding standardized surveillance (including broader participation in WHO’s GLASS), closing regional disparities in vaccine and diagnostic access, and embedding pandemic-preparedness lessons into routine respiratory infection control programs. A coordinated global approach integrating prevention, surveillance, innovation, and international collaboration will be essential to improve resilience against future respiratory outbreaks and to mitigate the long-term health and socioeconomic consequences of UATIs, particularly in underserved populations. 

## Figures and Tables

**Table 1:** Summary of the global burden, principal challenges, and priority interventions for upper airway tract infections (UATI).

Domain	Key Burden/Challenge	Priority Interventions
Epidemiology	~12.8 billion episodes/year; ~8 million DALYs; highest incidence in children <5y; marked LMIC excess in YLL	Standardized global/regional surveillance; disaggregated LMIC data collection
Antimicrobial resistance	Up to 40% of viral UATIs treated with unneeded antibiotics; rising GAS macrolide resistance; regional pneumococcal/H. influenzae resistance variation	Outpatient stewardship, rapid diagnostics, local resistance-informed prescribing
Vaccination	34% of LMICs have influenza vaccination policies vs. ~85% of HICs; no licensed GAS vaccine; vaccine hesitancy in HICs	Maternal RSV immunization, nirsevimab, PCV, GAS vaccine development, confidence-building campaigns
Economic burden	~US$40 billion/year in the USA; catastrophic out-of-pocket expenditure (>10% household income) in parts of Africa/Asia	Financial risk protection, subsidized primary care, sanitation investment
Future perspectives	Limited real-world validation of AI-assisted triage; uneven diagnostic access	Prospective validation of AI/predictive tools; equitable diagnostic scale-up; pandemic-preparedness integration

## Data Availability

Data reported are available for main literature online web database and registry.

## Abbreviations

The following abbreviations are used in this manuscript:

ACE2	Angiotensin-converting enzyme 2
AMR	Antimicrobial resistance
CDHR3	Cadherin-related family member 3
COVAX	COVID-19 Vaccines Global Access
DALY	Disability-adjusted life years
DTP3	Diphtheria-tetanus-pertussis 3
GBD	Global Burden of Disease
HRQoL	Health-related quality of life
HIC	High income countries
HIV	Human immunodeficiency virus
HRV	Human rhinovirus virus
ICAM1	Intercellular Adhesion Molecule 1
LMIC	Low- and middle-income countries
NPI	Non-Pharmaceutical Interventions
PVC	Pneumococcal conjugate vaccines
UATI	Upper airway tract infections
US	United States
WHO	World Health Organization
